# Physician Recruitment of Patients to Non-Therapeutic Oncology Clinical Trials: Ethics Revisited

**DOI:** 10.3389/fphar.2013.00025

**Published:** 2013-03-11

**Authors:** Lee Black, Gerald Batist, Denise Avard, Caroline Rousseau, Zuanel Diaz, Bartha Maria Knoppers

**Affiliations:** ^1^Centre of Genomics and Policy, McGill UniversityMontreal, QC, Canada; ^2^McGill University Centre for Translational Research in CancerMontreal, QC, Canada; ^3^Quebec-Clinical Research Organization in CancerMontreal, QC, Canada

**Keywords:** ethics, recruitment, clinical trial, informed consent, conflict of interest

## Abstract

Tailoring medical treatment to individual patients requires a strong foundation in research to provide the data necessary to understand the relationship between the disease, the patient, and the type of treatment advocated for. Non-therapeutic oncology clinical trials studying therapeutic resistance require the participation of patients, yet only a small percentage enroll. Treating physicians are often relied on to recruit patients, but they have a number of ethical obligations that might be perceived as barriers to recruiting. Concepts such as voluntariness of consent and conflicts of interest can have an impact on whether physicians will discuss clinical trials with their patients and how patients perceive the information. However, these ethical obligations should not be prohibitive to physician recruitment of patients – precautions can be taken to ensure that patients’ consent to research participation is fully voluntary and devoid of conflict, such as the use of other members of the research team than the treating physician to discuss the trial and obtain consent, and better communication between researchers, clinicians, and patients. These can ensure that research benefits are maximized for the good of patients and society.

## Introduction

There is a strong drive to provide personalized care in modern oncology practice (Schilsky, 2010). Tailoring medical treatment to individual patients, the centerpiece of the personalized medicine movement, requires a strong foundation in research to provide the data necessary to understand the relationship between the disease, the patient, and the type of treatment advocated for.

In cancer genomics, much of personalized medicine has turned toward therapeutic resistance: why are some patients resistant to targeted therapies despite expressing the druggable target? What are the mechanisms of acquired resistance and how can we circumscribe these? To answer these questions, traditional models of clinical trials cannot always be relied upon, as the intention is not to evaluate efficacy, but to give researchers clues as to how to improve treatment, either through rationale drug combinations or new drug designs. Many studies now require archived tumor tissue as entry criteria to a trial, or make use of banked tissue obtained through hospital biobanks to gain insight into the mechanism of action of the drug. In contrast, other studies ask that patients consent to a separate tissue procurement procedure for research purposes.

Especially for research into resistance mechanisms, many studies fall within this second category and must find participants who are willing to explicitly consent to biopsy-driven studies. This is exemplified in two trials led by the Quebec-Clinical Research Organization in Cancer (Q-CROC) on therapeutic resistance in colon cancer and breast cancer (clinicaltrial.gov identifier NCT00984048 and NCT01276899), and in many other studies of therapeutic resistance (Sequist et al., 2011; Doebele et al., 2012; Katayama et al., 2012; Shi et al., 2012). In these examples, patients consent to a non-therapeutic biopsy from a metastatic lesion that has progressed despite therapy. While the risks incurred are not high, they are more than minimal and without the expectation of clinical benefit to the patient from participation.

The need for patient participation in oncologic research is highlighted by the number of clinical trials requiring participants: over 12,000 listed by the National Cancer Institute alone (National Cancer Institute and National Institutes of Health, 2013). Some studies, though, have shown that only somewhere between 2 and 9% of all cancer patients actually enroll (Ross et al., 1999; Lara et al., 2001). For biopsy-driven studies without anticipated clinical benefit, we can expect the participation rate to decrease, although no formal evaluation has thus far been reported. The low participation in clinical trials delays the progression of research and the pursuit of personalized medicine.

One responsible party for at least broaching the subject of the patient’s participation in research is the patient’s treating physician. That physician is best placed to evaluate the patient’s physical condition, the social and mental status that might preclude the patient from participating in a trial, and the available avenues for both treatment and research. However, asking physicians to recruit their patients into research is not always a simple proposition. There are practical difficulties that inhibit patient recruitment such as time constraints, increased workload, and physician specialty and location (Lara et al., 2001; Klabunde et al., 2011; Kleiderman et al., 2012). Ethical obligations to promote the health and well-being of patients – and the treatment of disease as the highest priority – might also impact physicians’ decisions to bring up research with patients (Galvin et al., 2009; Kleiderman et al., 2012), as well as conflicts between these obligations and the duty to undertake research (Emanuel et al., 2008).

However, patient participation in research – a necessity for clinical advancements in personalized medicine – should be at least a consideration for physicians whose patients fit the criteria of a particular study. The evidence of patient willingness to participate (Agulnik et al., 2006) should allay some physician concerns, and the ethical ideal of autonomy (Beauchamp and Childress, 2009) should encourage at least informing patients of the research options available.

Yet, even given a willing physician and patient there are still complexities to the *ethical* *recruitment* of patients into research, especially when non-therapeutic procedures are involved. The goal of this paper is to examine two of these ethical complexities: voluntariness, a bedrock component of informed consent; and conflicts of interest, a long-term and consistent problem in biomedical research.

## Compromising the Voluntariness of Consent

The Nuremberg Code clearly states that research participation be undertaken “without the intervention of any element of force, fraud, deceit, duress, over-reaching, or other ulterior form of constraint or coercion…” (International Military Tribunal, 1949). This requirement of voluntariness has been confirmed universally over the years as an essential element of research participation (World Medical Association, 1964; Canadian Institutes of Health Research et al., 2010). However, a number of factors have the potential to compromise the voluntariness of consent by creating for the patient a risk-benefit perception that does not coincide with reality. These include the patient-physician relationship itself and a misunderstanding of the purposes of research (therapeutic misconception).

In an obvious challenge to voluntariness, a physician explicitly linking medical care to participation in research (e.g., “I won’t treat you unless you participate”) would oblige the patient to comply with the request of the physician. There are also subtler influences on the voluntary decision-making of patients. For example, the mere existence of the patient-physician relationship may cause patients to participate in a trial (Eggly et al., 2008). Indeed, patients are more likely to follow the suggestion of their physician because of the intimate relationship between the two and the dependency of the patient (Kass et al., 1996; Kleiderman et al., 2012). The saying “doctor knows best” has roots in reality, after all. If a patient is more likely to take a course of action because the physician is suggesting it (Ross et al., 1999), the voluntariness of that action will be questioned.

Therapeutic misconception can also affect patient perception of research risk and impact the decision to participate. This concept refers to “the notion that unless otherwise informed, research subjects will assume (especially, but not exclusively, in therapeutic research) that decisions about their care are being made solely with their benefit in mind” (Appelbaum et al., 1982). The presence of therapeutic misconception is common in research participants (Appelbaum et al., 2012; Pentz et al., 2012), and may be particularly problematic when participants are recruited by their own physician (de Melo-Martin and Ho, 2008; Kleiderman et al., 2012). Several factors can influence a patient’s response and comprehension, such as trust, (mis)understanding of the science, and knowing the difference between care and research (Kass et al., 1996; Lidz and Appelbaum, 2002). Importantly,

“[m]ost people have been socialized to believe that physicians (at least ethical ones) always provide personal care. It may therefore be very difficult, perhaps nearly impossible, to persuade subjects that this encounter is different, particularly if the researcher is also the treating physician, who has previously satisfied the subject’s expectations of personal care” (Appelbaum et al., 1987).

Thus, patients might assume that their physician would not suggest enrollment in research if it were not the best care. This is particularly true when all standard care has been administered and there remain limited treatment possibilities for the patient, or when the clinical research is presented as a treatment option (Miller and Rosenstein, 2003). However, even when a physician clearly emphasizes that the goal of research is *not* to provide care, as is frequently the case for biopsy-driven studies to explore therapeutic resistance, patients sometimes continue to believe that the research will provide them with direct benefits (Appelbaum et al., 1987).

Questions of voluntariness implicit in the patient-physician relationship and through therapeutic misconception do not assume that all patient decisions to participate in research are coerced, misinformed, or imposed: in research using adult patients, capacity, and a reliance on autonomy are generally presumed. To minimize factors that might compromise voluntariness of consent, candid physician conversations with patients are very important. The physician has a responsibility to distinguish between treatment and research and to clearly explain the implications for the patient.

## Conflicts of Interest

Incentives and rewards, both financial and non-monetary, have the potential to influence the recruitment of patients into research by both physicians and physician-investigators (Canadian Institutes of Health Research et al., 2010). These conflicts of interest are defined as “[t]he incompatibility of two or more duties, responsibilities, or interests … as they relate to the ethical conduct of research, such that one cannot be fulfilled without compromising another” (Canadian Institutes of Health Research et al., 2010). For example, physicians may receive funding to participate in research or to cover travel expenses. Additionally, non-monetary academic incentives can factor into conflicts of interest. Indeed, the avoidance of *anything* that could be considered a “conflict” is practically impossible: the pursuit of knowledge alone, as a clearly stated goal of biopsy-driven research, is in opposition to the personal interests of individual research participants. Therefore, the question becomes one of effect mitigation rather than prevention.

Certainly, some conflicts can and should be avoided, especially those pertaining to financial incentives (Bekelman et al., 2003; Wilson, 2010). A financial relationship between the research institution, study sponsor, and researcher is necessary to cover the direct costs associated with the research, but funds that directly benefit the researcher or physician (and to a lesser extent, academic institution) are most problematic. Thus, large payments to researchers, finder’s fees for physicians, and financial stakes in the company funding a researcher’s study raise obvious concerns of financial conflicts and are generally prohibited ethically and/or legally. At the very least, they must be disclosed to research institutions, ethics review boards, publishers, and, in many instances, to potential research participants (McCrary et al., 2000; Canadian Institutes of Health Research et al., 2010).

Conversely, other forms of conflict are more difficult to avoid in any academic research endeavor. Career advancement, professional accolades, and sustained research funding are incentives for physicians to undertake clinical research (Saver, 2012). As admitted by a researcher overseeing a gene therapy trial:

“To suggest that I acted or was influenced by money is really offensive to me…. I don’t think about how my doing this work is going to make me rich. It’s about leadership and notoriety and accomplishment. Publishing in first-rate journals. That’s what turns us on. You’ve got to be on the cutting edge and take risks if you’re going to stay on top” (Nelson and Weiss, 1999).

Thus, academic interests must be balanced with the benefits of research for the larger population of patients, and physicians have an obligation to remain objective regarding the care of their patients in light of the tensions between clinical treatment and medical research (Miller and Rosenstein, 2003). These more inherent and largely unavoidable conflicts in research are addressed through disclosure, ethics review, and other protections given to patients, especially in the initial contact for research recruitment.

## Some Solutions

Changes in research ethics over previous decades to address voluntariness of consent and conflicts of interest foresaw the very real dangers of physicians recruiting their own patients into research, even research in which they are not investigators but are merely serving a peripheral role. However, even when the research involves higher than minimal risk and no accompanying benefit for participants, this does not mean that patients are unapproachable.

The performance of non-therapeutic research, like other human-subject research, requires the consideration of all stakeholders. Patients, clinicians, researchers, and research nurses and coordinators all have different perspectives that should be examined prior to including patients in the research. In a recent pilot study by Kleiderman et al. clinicians, research nurses and coordinators, and researchers were asked about the barriers they perceived in recruiting patients into non-therapeutic research as well as any facilitators that aided in this recruitment. The paper concluded that ethical risks are important considerations when recruiting patients but that there are “mechanisms to ensure that they are appropriately informed of the risks…” (Kleiderman et al., 2012). The solutions we suggest below are geared to promote an informed consent that minimizes the ethical risks discussed above.

Physicians are possibly in the best position to inform patients of the existence of studies that might interest or benefit them due to their inherent knowledge of patients’ health conditions. However, commentators have pointed out that physicians – especially when not intimately involved with the research – often do not fully understand the studies they are proposing to patients, especially the potential harms and benefits (Daugherty et al., 1995). Physicians should therefore limit their role to that of information gateway to other health professionals involved in the research, such as research nurses, research coordinators, or other physicians (Kleiderman et al., 2012). This could partly absolve physicians of many of the ethical hurdles they otherwise face. An examination of these restrictions in the context of the two themes discussed above – voluntariness and conflicts of interest – demonstrates the advantages of a limited recruitment role for treating physicians.

Voluntary consent will always be an issue in any kind of research recruitment. However, if a person other than the treating physician explains the non-therapeutic nature of the research in detail and obtains patient consent, it will reduce the potential for undue influence of the physician on patient participation. Likewise, conflict of interest can be limited by avoiding situations when it is otherwise possible. Using other members of the research team to actively recruit and inform patients, and to sign or co-sign consent forms, will reduce both real and perceived conflicts pertaining to recruitment.

The use of professionals other than the researcher or patient’s physician – a neutral third-party – to initiate and maintain contact with the patient/potential participant is not a novel idea. The investigator in the case of Jesse Gelsinger, who died during a gene therapy trial, recognized that

The scientists behind the technology believe in the potential of the technology and pursue its development with zeal…. The crux of the problem is to assure that the subject receives a balanced and unbiased view of the risks and benefits of his/her participation in the trial and that s/he can make decisions without influence or concern over negative consequences (Wilson, 2009).

Many researchers, as well as the investigating authorities examining Gelsinger’s death, acknowledge the benefits of a third-party providing potential participants with unbiased information (Wilson, 2010). In addition, the use of patient advocates to assist in study and consent design has been suggested as another means to improve transparency and public trust in research (Katz et al., 2012). These individuals may have a more patient-centered perspective than members of the research team. Unfortunately, although the use of someone outside of the patient-physician relationship to aid in patient recruitment is suggested as an ethically appropriate solution, there has been little data demonstrating the effectiveness of this in limiting ethical risks.

We must also point out that the use of a neutral third-party in the patient recruitment process may imply a limitation on patient contact with their physician. We do not believe that this is a necessary correlation, and a rigid wall between a patient and treating physician is not required. Certainly, physicians’ involvement in recruiting their own patients into research raises ethical concerns, but the patient-physician relationship need not end. Indeed, the patient may feel more comfortable knowing that his or her physician is involved in the research. The use of a third-party to gain consent for participation is intended to reduce potential harms to the patient’s autonomy represented by the physician’s involvement in the research, not infringe upon the patient-physician relationship.

## Conclusion

The need for patient participation in research to find personalized solutions to medical conditions and to understand mechanisms of resistance will only increase as scientific knowledge and technology advance. In the drive to find these participants, physicians will be relied upon because of their familiarity with their patients’ conditions, as well as information that would advise against patient involvement, including both physical and emotional considerations.

The above discussion demonstrates that physicians do not have to remove themselves from the recruitment process. Although voluntariness of consent and conflicts of interest must be monitored in any research enterprise, these ethical considerations should not result in a blanket prohibition on physicians recruiting or talking about research involvement with their patients. Limited involvement, such as mentioning the existence of a study and then referring the patient to a member of the clinical research team or a patient advocate, is one way to mitigate any ethical concerns. In fact, it is not realistic to prohibit physicians from discussing potential clinical trial participation to their patients, especially if we want research to advance on the very conditions affecting patients and considering that patients may count on their physician to be aware of ongoing research, whether therapeutic on non-therapeutic.

It should be noted that these ethical constraints on participant recruitment are important in any form of medical research, not just within the context of the studies we focus on in this article. Although recruiting patients into non-therapeutic research with more than minimal risk may raise the ethical stakes, similar considerations of voluntariness and conflicts of interest are underlying currents across all human-subject research.

In order to address these ethical considerations for patient recruitment into clinical trials, there is a need for better intra-professional communication about available trials, eligibility criteria, and other relevant information, as well as further research regarding physicians’ needs when communicating with patients. In the meantime, by limiting the involvement of treating physicians in the recruitment process we can minimize ethical risks to achieve greater transparency and professional integrity, so that research benefits can be maximized for the good of patients and society.

## Conflict of Interest Statement

The authors declare that the research was conducted in the absence of any commercial or financial relationships that could be construed as a potential conflict of interest.
